# Reversion and conversion of *Mycobacterium tuberculosis *IFN-γ ELISpot results during anti-tuberculous treatment in HIV-infected children

**DOI:** 10.1186/1471-2334-10-138

**Published:** 2010-05-27

**Authors:** Tom G Connell, Mary-Ann Davies, Christine Johannisen, Kathryn Wood, Sandy Pienaar, Katalin A Wilkinson, Robert J Wilkinson, Heather J Zar, David Beatty, Mark P Nicol, Nigel Curtis, Brian Eley

**Affiliations:** 1 Institute of Infectious Diseases and Molecular Medicine, University of Cape Town, Cape Town, South Africa; 2 Department of Paediatrics, The University of Melbourne, Australia; 3 Infectious Diseases Unit, Department of General Medicine, Australia; 4 Murdoch Children's Research Institute; Royal Children's Hospital Melbourne, Parkville, Australia; 5 Red Cross Children's Hospital and School of Child and Adolescent Health, University of Cape Town, Cape Town, South Africa; 6 School of Public Health and Family Medicine, University of Cape Town, Cape Town, South Africa; 7 National Institute for Medical Research, Mill Hill, London NW7 1AA, UK; 8 Division of Medicine, Imperial College London, London, W2 1PG, UK; 9 Division of Medical Microbiology, University of Cape Town and National Health Laboratory Service, Cape Town, South Africa

## Abstract

**Background:**

Recent interest has focused on the potential use of serial interferon gamma (IFN-γ) release assay (IGRA) measurements to assess the response to anti-tuberculous (TB) treatment. The kinetics of IFN-γ responses to *Mycobacterium tuberculosis *(MTB) antigens in HIV-infected children during treatment have not however been previously investigated.

**Methods:**

IFN-γ responses to the MTB antigens, ESAT-6, CFP-10 and PPD were measured by an enzyme-linked immunospot assay (IFN-γ ELISpot) at presentation and at one, two and six months after starting anti-tuberculous treatment in HIV-infected children with definite or probable TB. Responses at different time points were compared using a Mann-Whitney U test with paired data analysed using the Wilcoxon signed rank test. A Fisher's exact or Chi-squared test was used to compare proportions when test results were analysed as dichotomous outcomes.

**Results:**

Of 102 children with suspected TB, 22 (21%) had definite TB and 24 (23%) probable TB. At least one follow up IFN-γ ELISpot assay result was available for 31 (67%) of the 46 children. In children with definite or probable TB in whom the IFN-γ ELISpot assay result was positive at presentation, anti-tuberculous treatment was accompanied by a significant decrease in both the magnitude of the IFN-γ response to individual or combined MTB-specific antigens (ESAT-6 median 110 SFCs/10^6 ^PBMC (IQR 65-305) at presentation vs. 15 (10-115) at six months, p = 0.04; CFP-10 177 (48-508) vs. 20 (5-165), p = 0.004, ESAT-6 or CFP-10 median 250 SFCs/10^6 ^PBMC (IQR 94-508) vs. 25 (10-165), p = 0.004) and in the proportion of children with a positive IFN-γ ELISpot assay (Fisher's exact test: ESAT-6 15/0 vs 5/11, p = 0.0002, CFP-10 22/0 vs 8/17, p = 0.0001, ESAT-6 or CFP-10 22/0 vs. 9/17, p= 0.002). However almost half of the children had a positive IFN-γ ELISpot assay after six months of anti-tuberculous treatment. In addition, there was conversion of the IFN-γ ELISpot assay result during anti-tuberculous therapy in six of 12 children in whom the initial IFN-γ ELISpot assay was negative.

**Conclusions:**

In HIV-infected children with definite or probable TB, anti-tuberculosis treatment is accompanied by a reduction in the magnitude of the IFN-γ ELISpot response to MTB-antigens. However, serial IFN-γ ELISpot measurements appear to have limited clinical utility in assessing a successful response to anti-tuberculous treatment in HIV infected children.

## Background

Interferon gamma (IFN-γ) release assays (IGRA) based on the *in vitro *T cell responses to *Mycobacterium-tuberculosis *(MTB)-specific antigens have the potential to improve the diagnosis of tuberculosis (TB) [1]. An increasing body of evidence, predominantly in immunocompetent adult populations, shows that IGRA (either whole blood based (for example, QuantiFERON-TB Gold assay) or enzyme-linked immunospot (IFN-γ ELISpot), (for example, T.SPOT.*TB *assay) have a similar sensitivity to that of the tuberculin skin test (TST) (between 70-90%) and a higher specificity for the detection of active TB disease [2]. IGRA have also been shown to have higher sensitivity compared to TST for the detection of active TB disease in HIV-infected persons, with the IFN-γ ELISpot appearing to be superior to QuantiFERON-TB Gold in this setting [3].

There is much less data on the use of either IGRA in children and the sensitivity of the IFN-γ ELISpot and QuantiFERON-TB Gold for the detection of active TB disease [4,5] and latent TB infection [6,7] in young children has been questioned [8-11]. However, in HIV-infected children, in whom the performance of the TST is impaired, studies have shown an IFN-γ ELISpot to have higher sensitivity than the TST for the detection of active TB disease [12,13].

In addition to their use as an adjunctive test for the diagnosis of active TB disease in adults and children, emerging data suggest a potential role for IGRA in monitoring the response to anti-tuberculous treatment. Animal and human studies have shown a relationship between the mycobacterial bacillary load and the magnitude of IFN-γ responses to MTB-antigens [14,15]. It has therefore been postulated that a decrease in the magnitude of IFN-γ responses to MTB-specific antigens can be used as a surrogate marker of treatment success [16]. However, studies using serial QuantiFERON-TB Gold or IFN-γ ELISpot assays measurements in adults taken during treatment of either latent TB infection [17-19] or active TB disease [14,20-30] in various epidemiological settings have shown conflicting results with IFN-γ responses decreasing [17,18,20,22,25,26], increasing [23,24,29,30] or remaining unchanged [21,28] in response to treatment.

Only three studies have investigated the kinetics of IFN-γ responses to MTB-specific antigens in children (two with an IFN-γ ELISpot and one with QuantiFERON-TB Gold In Tube assay) [31-33] and no study has investigated IFN-γ responses to MTB-antigens during anti-tuberculous treatment in HIV-infected children.

As part of a prospective study evaluating the usefulness of an IFN-γ ELISpot assay in HIV-infected children with suspected TB [12], we investigated the kinetics of T cell responses to MTB-antigens in a subset of HIV-infected children with definite or probable TB during anti-tuberculous treatment. We hypothesized that successful anti-tuberculous treatment would be accompanied by a reduction in the magnitude of the IFN-γ ELISpot response to MTB-antigens in HIV-infected children with definite or probable TB that may result in reversion (from positive to negative) of the IFN-γ ELISpot assay. The aim of the study was to determine the potential clinical utility of serial IFN-γ ELISpot assay measurements to assess the response to treatment in HIV-infected children in a high TB incidence area.

## Methods

### Patients

The study was approved by the University of Cape Town Research Ethics committee (Rec No. 451/2005). The study population comprised the 22 children with definite TB and 24 children with probable TB identified from 102 HIV-infected children with suspected TB in a previously published study [12]. Written witnessed informed consent was obtained from a parent in their preferred language. All children were prospectively recruited from Red Cross Children's Hospital, Cape Town, South Africa (estimated TB incidence rate 1600/100,000) [34]) between April 2006 and May 2007. HIV-infected children were classified as having definite or probable TB based on a combination of well-defined clinical and microbiological criteria [12]. Definite TB was defined as isolation of *M. tuberculosis *from culture or the detection of acid-fast bacilli on microscopy of an appropriate site-specific clinical sample. Probable TB was defined as symptoms suggestive of TB and at least two of the following: TB contact, chest radiograph findings consistent with TB and good response to TB treatment. An IFN-γ ELISpot assay was done prior to, and after one, two and six months of anti-tuberculous treatment.

The IFN-γ ELISpot assay was performed as previously described [12]. Briefly, peripheral blood mononuclear cells were stimulated with early secretory antigen target-6 (ESAT-6; a pool of 15-mer peptides overlapping by 10 amino acids, Peptide Protein Research UK; final concentration 5 μg/ml/peptide), culture filtrate protein 10 (CFP-10, a pool of 15-mer peptides overlapping by 10 amino acids, Peptide Protein Research, UK; final concentration 5 μg/ml/peptide) and purified protein derivative (PPD, Evans Vaccines PL00039/0439, final concentration 200 units/ml). No antigen was added to the negative control well. Anti CD3 mAb CD3-2 (Mabtech) at a final concentration of 100 ng/ml was included as a positive control. Mean IFN-γ responses to each of the antigens and controls were calculated after subtraction of background IFN-γ responses obtained from the negative control wells. IFN-γ responses were expressed as spot forming cells (SFCs)/million (10^6^) PBMC. The IFN-γ ELISpot was considered *positive *if the number of SFCs in the antigen-stimulated wells was greater than or equal to 5 SFCs/2 × 10^5 ^(25 SFCs/10^6^) above the background response, and, in cases where the background response was greater than or equal to 10 SFCs, more than twice the background response.

### Statistical analysis

Data were analysed using Prism Graphpad v5 (Graphpad software, Inc, San Diego, California, USA). The Mann-Whitney U test was used to compare nonparametric unpaired data. The median number of SFCs at each time point was compared using the Kruskal-Wallis test. Paired data were compared using the Wilcoxon signed rank test. A Fisher's exact or Chi-squared test was used to compare proportions when test results were analysed as dichotomous outcomes.

## Results

The demographic details of the patients included in the study are shown in Table 1. Of the 22 children with definite TB, MTB culture was positive in 20 (91%). In the two remaining children AFB were seen on site-specific clinical specimens but cultures were negative. At diagnosis, 12 (26%) children with definite or probable TB were on anti-retroviral treatment (ART). A further eighteen (39%) children commenced ART during the study period.

**Table 1:** Demographic details for the patients included in the study (n = 46)

Median age in months (IQR)	22.6 (12.4 - 59.6)
Male n(%)	30 (65)
Median weight for age score (IQR)	-2.3 (-3.3 - -1.4)
BCG scar present n (%)	33 (72)
Localisation of TB	
Pulmonary only	42 (91)
Extrapulmonary TB	1 (2)
Disseminated TB	3 (7)
Median TST induration (range) mm	0 (0 - 11)
Median CD4 (IQR) %	20.2 (12.4 - 28.3)
Median absolute CD4 count (IQR) cells/ml	712 (392 - 1365)
On anti-retrovirals at diagnosis n (%)	10 (22)

**Figure 1:**
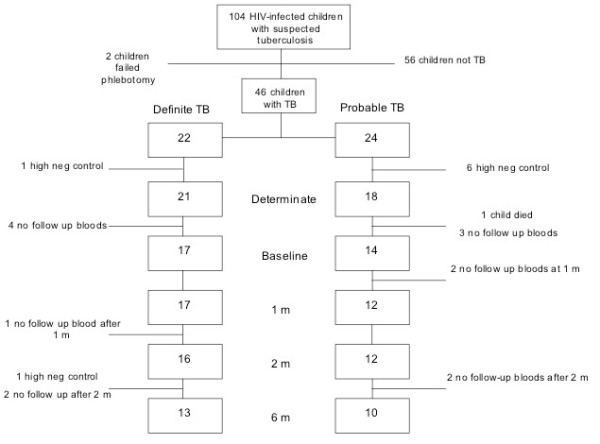
Flow chart of recruitment and follow up

At least one follow up IFN-γ ELISpot assay result was available for 31 (67%) of the 46 children with definite or probable TB. The number of children with definite or probable TB that had a repeat IFN-γ ELISpot at each of the three time points is shown in Figure 1. Of the 22 children with definite TB, 21 (95%) had an interpretable IFN-γ ELISpot assay result. Of these 21 children, 15 (71%) had a positive response to at least one of the MTB-antigens ESAT-6, CFP-10 or PPD at presentation and 17 (81%) children returned for at least one follow up IFN-γ ELISpot assay during anti-tuberculous treatment. Of the 24 children with probable TB, 18 (75%) had an interpretable IFN-γ ELISpot assay result. Of these 18 children, 12 (75%) had a positive response to at least one of the MTB-antigens ESAT-6, CFP-10 or PPD at presentation and 14 (58%) children returned for at least one follow up IFN-γ ELISpot assay during anti-tuberculous treatment. Of the six children with probable TB whose initial IFN-γ ELISpot assay yielded an indeterminate result, two had a persistently elevated background IFN-γ response that rendered the IFN-γ ELISpot assay result indeterminate on at least two follow up time points.

At presentation, there was no difference in the proportion of children with a positive IFN-γ ELISpot between those who had received prior TB treatment and those who had not (5/11 vs. 20/28, p = 0.15). In children with definite or probable TB, there was no difference in the median (interquartile range) CD4% between children with a positive IFN-γ ELISpot compared to those with a negative IFN-γ ELISpot (positive 19.12 (10.0-35.5) vs negative 19.7 (13.6-31.1), p = 0.78).

**Figure 2:**
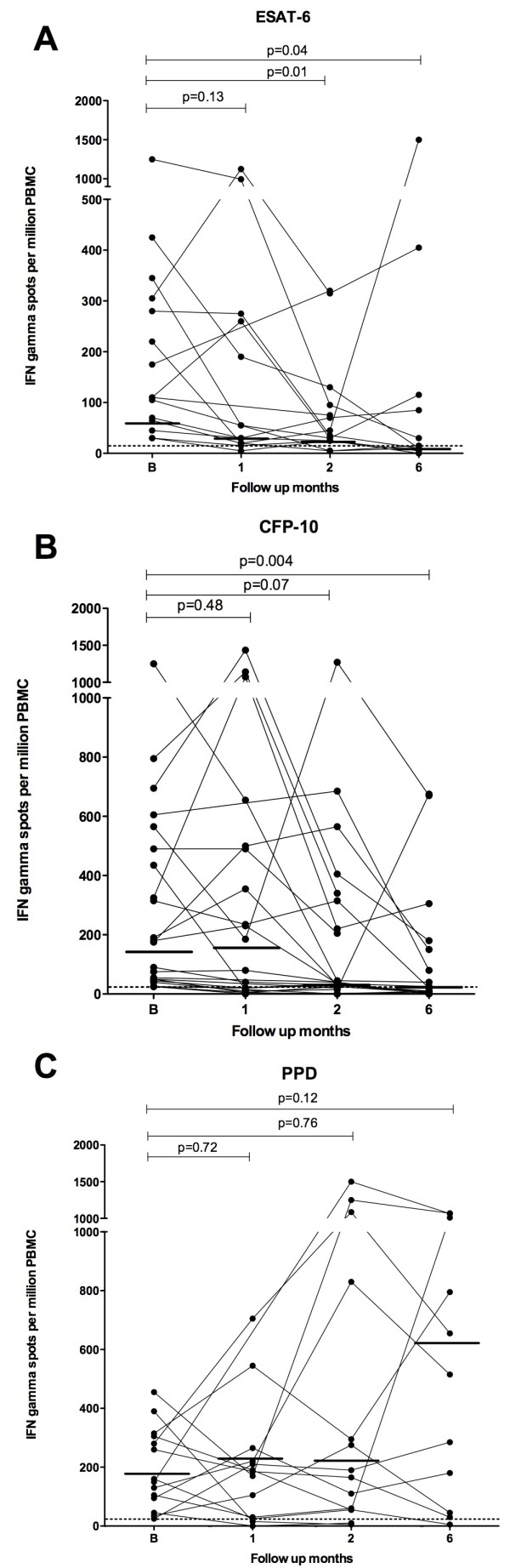
**IFN-γ responses to ESAT-6 (A), CFP-10 (B) and PPD (C) in HIV-infected children in whom the ELISpot was positive at presentation at one, two and six months**. P values represent the difference in median IFN-γ responses (indicated by horizontal solid bar) at the different follow up points.

In the group of children with definite or probable TB who had a positive IFN-γ ELISpot at presentation, there was a significant change in median IFN-γ responses to MTB-antigens during anti-tuberculous treatment (Table 2 and Figure 2 Panel A, B, C). Specifically, there was a decrease in median IFN-γ responses to ESAT-6 and CFP-10 and an increase in the response to PPD following six months of anti-tuberculous treatment, though the change in response to PPD failed to reach statistical significance (ESAT-6 median 110 SFCs/10^6 ^PBMC (IQR 65-305) vs. 15 (10-115), p = 0.04; CFP-10 177 (48-508) vs. 20 (5-165), p = 0.004; PPD 155 (82-307) vs. 515 (45-1010), p = 0.12). In addition, there was a decrease in the highest median IFN-γ response to either antigen (ESAT-6 or CFP-10) following six months of anti-tuberculous treatment (highest response to either ESAT-6 or CFP-10 median 250 SFCs/10^6 ^PBMC (IQR 94-508) vs. 25 (10-165), p = 0.004).

**Table 2:** IFN- γ ELISpot assay results during anti tuberculous treatment in HIV-infected children

Antigen	Baseline	1 month	2 months	6 months	
ESAT-6 (n = 15)*					
Median (IQR) IFN-γ response (SFCs/10^6 ^PBMC)	110 (65-305)	55 (20-267)	35 (25-95)	15 (10-115)	p = 0.04^†^
No (%) children with a positive ELISpot result	15 (100)	9/13 (69)	13/15 (87)	5/11 (45)	p = 0.006^ϕ^
No (%) children with a reduced IFN- γ response compared to baseline		11/13 (85)	12/15 (80)	7/11 (64)	NA

					

CFP-10 (n = 22)*					
Median (IQR) IFN-γ response (SFCs/10^6 ^PBMC)	177 (48-508)	185 (15-500)	40 (27-327)	20 (5-165)	p = 0.004^†^
No (%) children with a positive ELISpot result	22 (100)	13/19 (68)	17/21 (81)	8/17 (47)	p = 0.001^ϕ^
No (%) children with a reduced IFN-γ response compared to baseline		11/19 (55)	16/21 (76)	14/17 (82)	NA

					

ESAT-6 or CFP-10 (n = 22)*					

Median (IQR) IFN-γ response (highest response to either)	250 (94-508)	190 (20-500)	95 (10-115)	25 (10-165)	p = 0.004^†^

No (%) children with a positive ELISpot result	22 (100)	14/19 (74)	19/21 (90)	9/17 (53)	p = 0.002^ϕ^
No (%) children with a reduced IFN-γ response ***to either antigen ***compared to baseline		14/19 (74)	16/21 (76)	14/17 (82)	NA

					

PPD (n = 14)*					
Median (IQR) IFN-γ response	155 (82-307)	185 (27-242)	177 (55-893)	515 (45-1010)	p = 0.12^†^
No (%) children with a positive ELISpot result	14 (100)	10/13 (77)	12 (86)	10/11(91)	p = 0.56^ϕ^
No (%) children with a reduced IFN-γ response compared to baseline		6/13 (46)	7/14 (50)	1/11 (9)	NA

* Children with definite TB or probable TB who had a positive IFN-γ ELISpot at presentation

† p value indicates difference in magnitude of the IFN-γ ELISpot responses at baseline vs. 6 months (Mann Whitney U test)

ϕ p value indicates difference in the proportion of children with a positive IFN-γ ELISpot assay (Chi Square test)

Paired IFN-γ responses to individual antigens were analysed at the different time points during anti-tuberculous treatment to investigate the within patient kinetics of IFN-γ responses. In children with definite or probable TB and a positive IFN-γ ELISpot at presentation, paired analysis of IFN-γ responses showed a statistically significant increase in IFN-γ responses to PPD at six months (p = 0.02), decrease in IFN-γ responses to ESAT-6 at two months (p = 0.01) and decrease in IFN-γ responses to CFP-10 at two (p = 0.03) and six months (p = 0.02).

In children with definite or probable TB and a positive IFN-γ ELISpot at presentation, there was a decrease in the proportion of children who had a positive IFN-γ response to individual antigens ESAT-6 and CFP-10 following six months of anti-tuberculous treatment (Chi square for trend: ESAT-6 p = 0.006, CFP-10 p = 0.001). Furthermore, there was a decrease in the proportion of children who had a positive IFN-γ response to either antigen (ESAT-6 or CFP-10, 22/0 vs. 9/17, p= 0.002). However, almost half of children had a positive IFN-γ ELISpot response to ESAT-6 (5/11 (45%)), CFP-10 (8/17 (47%)) or either ESAT-6 or CFP-10 (9/17 (53%) following six months of anti-tuberculous treatment. The proportion of children that had a positive IFN-γ ELISpot response to PPD remained unchanged during treatment (Table 1).

**Figure 3:**
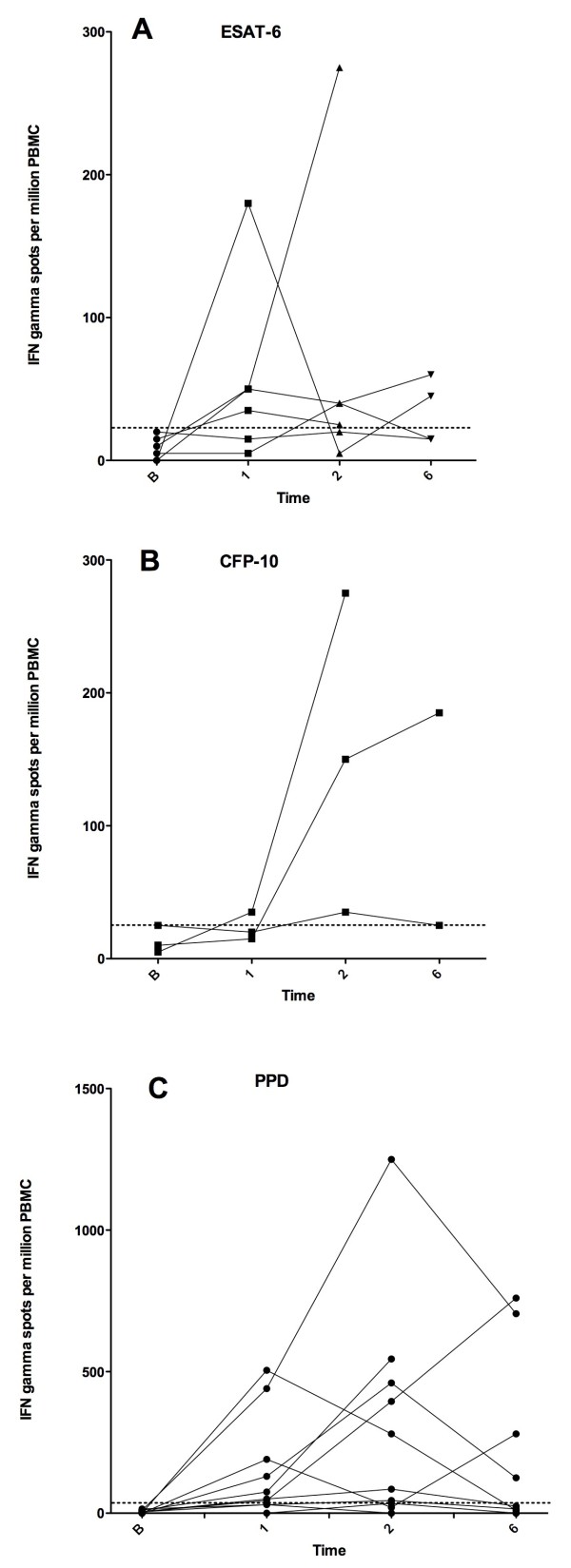
**IFN-γ responses to ESAT-6 (A) CFP-10 (B) and PPD (C) in children with a initial negative response that became positive at some point during follow up**.

Of 12 children with definite or probable TB that were IFN-γ ELISpot negative at presentation, 6 (50%) became IFN-γ ELISpot positive (i.e. converted rather than reverted) at one point or more during anti-tuberculosis treatment, the rest remaining persistently IFN-γ ELISpot negative during follow-up. The IFN-γ responses at various time points during anti-tuberculous treatment in these children are shown in Figure 3 (Panel A, B and C).

## Discussion

Our study is the first to describe the kinetics of IFN-γ responses to MTB-antigens during anti-tuberculous treatment in HIV-infected children. We have shown that, in HIV-infected children with definite or probable TB in whom the result of an IFN-γ ELISpot assay is positive at presentation, anti-tuberculous treatment is accompanied by a significant decrease in both the magnitude of the IFN-γ response to MTB-antigens and in the proportion of children with a positive ESAT-6 or CFP-10 response following six months of anti-tuberculous treatment. We have also shown that in some children, anti-tuberculous treatment is accompanied by a transient increase in the IFN-γ ELISpot to MTB-antigens. The clinical usefulness of serial IFN-γ ELISpot measurements for assessing a successful curative response to anti-tuberculous treatment in this setting is, however, questionable as almost half of the children had a positive IFN-γ ELISpot assay result at the completion of anti-tuberculous treatment.

A small number of previous studies in HIV-uninfected children have provided preliminary data on the effect of anti-tuberculous treatment on IFN-γ responses to MTB-antigens in young children [31-33]. In a cohort of South African children Nicol *et al *found an increased IFN-γ ELISpot response to PPD one month after starting anti-tuberculous treatment followed by a subsequent decline after three and six months [33]. In a more recent study in French children, Hermann *et al *reported a decrease in the magnitude of IFN-γ responses (measured using QuantiFERON-TB Gold In Tube (QFT-GIT)) during anti-tuberculous treatment but found no difference in the proportion of children that had a positive assay result at the completion of treatment compared to baseline [32]. Specifically, of 32 children with active TB, 25 had a QFT-GIT at baseline and 6 months. Although there was a reduction in the magnitude of the IFN-γ ELISpot response to MTB-specific antigens in these children with treatment, the number (proportion) of children with a positive QFT-GIT assay result was not significantly different (21/25 (84%) at baseline vs. 18/25 (72%) at 6 months, p = 0.5).

In a TB contact investigation, Ewer *et al *documented a decrease in the magnitude of IFN-γ ELISpot responses to ESAT-6 and CFP-10 in 38 TST-positive adolescents following three months of TB preventive treatment of latent TB infection. In this study, 32 (84%) adolescents remained IFN-γ ELISpot positive 15 months beyond the completion of treatment [31].

The findings of our study in HIV-infected children and the studies in HIV-uninfected children described above are consistent with data in similar studies in adults. These studies indicate that the kinetics of IFN-γ responses to MTB antigens during anti-tuberculous treatment are not consistent. Of the studies that have reported serial IGRA (whole blood or IFN-γ ELISpot assay) measurements prior to, during or at the completion of treatment of active TB disease in adults, four have shown an increase [23,24,29,30] and six a decrease [20,22,25-27,35] in the magnitude of the IFN-γ responses. For example, Ferrand *et al *investigated the IFN-γ ELISpot response to ESAT-6 at presentation, during and at the completion of anti-tuberculous treatment in patients with pulmonary TB [24]. In this study IFN-γ ELISpot responses to ESAT-6 were higher at the completion of anti-tuberculous treatment than at presentation. Similarly, Ulrichs *et al *documented the IFN-γ ELISpot response to ESAT-6 in 10 adult patients with pulmonary TB prior to and 60 days after starting anti-tuberculous treatment [29]. In these 10 patients, the median IFN-γ ELISpot response to ESAT-6 was significantly higher at 60 days compared to baseline IFN-γ responses.

In contrast, Aiken et al documented a reduction in the magnitude of the IFN-γ ELISpot response to ESAT-6 and CFP-10 respectively in response to anti-tuberculous treatment in almost 80% of patients with culture proven tuberculosis [20]. However, in agreement with the findings from our study, over half of the patients in this study had a positive IFN-γ ELISpot assay result at the completion of anti-tuberculous treatment. In an Indian study, Katiyar *et al *investigated the kinetics of the IFN-γ responses to ESAT-6, CFP-10 and TB 7.7 during anti-tuberculous treatment in 79 adults with confirmed TB by repeating a QFT-GIT assay at two and 6 months [25]. In this study, there was a progressive reduction in the magnitude of the IFN-γ response during anti-tuberculous treatment. The number (proportion) of patients with a positive QFT-GIT assay result at presentation, two and 6 months was 72 (91%), 46 (59%) and 37 (47%) respectively. In another study in adults, Carrara *et al *documented a decrease in the IFN-γ ELISpot response to ESAT-6 following three months of anti-tuberculous treatment in 13 of 18 patients with culture confirmed TB that had a favourable response to treatment [22]. Notably, in the five remaining patients, all of whom were persistently culture positive at three months, a reduction in the magnitude of the IFN-γ ELISpot response to ESAT-6 was observed but the IFN-γ ELISpot assay result remained positive. Although various epidemiological factors may have influenced the results of these longitudinal studies in adults, the most consistent finding is a reduction in the magnitude of the IFN-γ response to MTB-antigens during anti-tuberculous treatment. However in many cases, this reduction does not result in a reversion of the IGRA result.

In the current study, almost one third of children with confirmed TB had a negative IFN-γ ELISpot at presentation. Despite the reported sub-optimal sensitivity of IGRA in children, we previously reported that approximately two-thirds of HIV-infected children with smear negative TB are IFN-γ ELISpot positive at presentation [12]. Our study suggests that with successful treatment (clinical improvement and weight gain at follow up visits), children who have a positive IFN-γ ELISpot response at presentation show reduced IFN-γ responses to the MTB-specific antigens at each follow-up time point. An early reduction in IFN-γ responses to MTB-specific antigens may provide a measure of reassurance to the treating physician and would be of practical relevance in a child co-infected with HIV and TB in whom assessing the clinical response to treatment may be problematic. In contrast, identifying children in whom IFN-γ responses remain persistently high or in whom responses increase, may alert the clinician earlier to the possibility of treatment failure as a result of drug-resistant TB or poor adherence to treatment. In our study, we observed one patient to be persistently culture positive two months into treatment. This was accompanied by a rapid increase in the IFN-γ response to CFP-10 (data not shown).

In agreement with the findings in our study, IFN-γ responses to PPD have been reported to increase during anti-tuberculous treatment in other studies. The reason behind this is currently unknown. Several authors have reported interesting differential decay kinetics to the individual MTB-specific antigens ESAT-6 and CFP-10. In a large study in Singapore, a significant reduction in median IFN-γ responses to CFP-10, but not ESAT-6 was found following six months of isoniazid preventive treatment [17]. Similarly, in another study, a significant reduction in IFN-γ responses to CFP-10 was found in 40 patients with active TB disease during anti-tuberculous treatment but there was no change in IFN-γ responses to ESAT-6 [26].

In our study, we observed an apparent increase in IFN-responses to MTB-antigens from baseline at some point during anti-tuberculous treatment (resulting in a conversion from a negative to positive IFN-γ ELISpot assay result) in 12 children with definite or probable TB. This may be the result of ongoing MTB exposure or may illustrate the complexity of the underlying dynamic immune response at different time points during anti-tuberculous treatment.

There are a number of limitations to our study. Firstly, the number of children with serial IFN-γ ELISpot results at each of the different time points was relatively small. Despite this, we were able to identify definite trends in MTB-specific immune responses during anti-tuberculous treatment. Secondly, we were unable to assess the influence of concomitant anti-retroviral treatment (ART) on IFN-γ ELISpot responses, as the study was not designed to explore this in any detail. However, only a minority of the children were on ART at diagnosis. Finally, the IFN-γ ELISpot assay used in the study, though similar to the commercial T.SPOT.*TB *assay, incorporating pools of peptides of ESAT-6 and CFP-10 as stimulatory antigens, was not a commercial version of the assay. However, a similar assay to the one used in this study, has been used in other published studies [33,36].

## Conclusion

We have shown that in HIV infected children with definite or probable TB in whom an IFN-γ ELISpot assay is positive at presentation, anti-tuberculous treatment is accompanied by a reduction in IFN-γ responses to MTB-specific antigens. However, the clinical usefulness of serial IFN-γ ELISpot measurements to assess a successful response to anti-tuberculous treatment in HIV-infected children in a high TB incidence area appears limited.

## Abbreviations

ESAT-6: Early secreted antigenic target-6; CFP-10: Culture filtrate protein-10; ELISpot: Enzyme linked immunospot; IFN-γ: Interferon gamma; MTB: *Mycobacterium tuberculosis*; IGRA: Interferon gamma release assays; PPD: Purified protein derivative

## Competing interests

The authors declare that they have no competing interests.

## Author' contributions

MAD, TGC, RJW, KAW, NC, HZ, BE, DB and MPN conceived and designed the study. MAD, TGC, HZ, BE and CJ enrolled patients. MAD, TGC and CJ performed phlebotomy for the immunological assays. DB, MPN, MAD, BE, RJW and KAW contributed reagents/materials. MAD, TGC and CJ examined the patients at follow-up visits. KW and SP performed IFN-γ ELISpot assays. TGC, MAD, NC, RJW, KAW, HZ, BE and MPN wrote the final manuscript. All authors have read and approved the final manuscript.

## Pre-publication history

The pre-publication history for this paper can be accessed here:

http://www.biomedcentral.com/1471-2334/10/138/prepub

## References

[B1] Andersen P Munk ME Pollack JM Doherty MT Specific immune-based diagnosis of tuberculosis The Lancet 2000 356 1099 1104 10.1016/S0140-6736(00)02742-2 11009160

[B2] Menzies D Pai M Comstock G Meta-analysis: new tests for the diagnosis of latent tuberculosis infection: areas of uncertainty and recommendations for research Ann Intern Med 2007 146 340 354 17339619 10.7326/0003-4819-146-5-200703060-00006

[B3] Rangaka MX Diwakar L Seldon R van Cutsem G Meintjes GA Morroni C Mouton P Shey MS Maartens G Wilkinson KA Clinical, immunological, and epidemiological importance of antituberculosis T cell responses in HIV-infected Africans Clin Infect Dis 2007 44 12 1639 1646 10.1086/518234 17516410

[B4] Kampmann B Whittaker E Williams A Walters S Gordon A Martinez-Alier N Williams B Crook AM Hutton AM Anderson ST Interferon-gamma release assays do not identify more children with active tuberculosis than the tuberculin skin test Eur Respir J 2009 33 6 1374 1382 10.1183/09031936.00153408 19196815

[B5] Nicol MP Davies MA Wood K Hatherill M Workman L Hawkridge A Eley B Wilkinson KA Wilkinson RJ Hanekom WA Comparison of T-SPOT.TB assay and tuberculin skin test for the evaluation of young children at high risk for tuberculosis in a community setting Pediatrics 2009 123 1 38 43 10.1542/peds.2008-0611 19117858

[B6] Connell TG Curtis N Ranganathan SC Buttery JP Performance of a whole blood interferon gamma assay for detecting latent infection with Mycobacterium tuberculosis in children Thorax 2006 61 7 616 620 10.1136/thx.2005.048033 16601088 PMC2104654

[B7] Connell TG Ritz N Paxton GA Buttery JP Curtis N Ranganathan SC A three-way comparison of tuberculin skin testing, QuantiFERON-TB gold and T-SPOT.TB in children PLoS ONE 2008 3 7 e2624 10.1371/journal.pone.0002624 18612425 PMC2440545

[B8] Haustein T Ridout DA Hartley JC Thaker U Shingadia D Klein NJ Novelli V Dixon GL The likelihood of an indeterminate test result from a whole-blood interferon-gamma release assay for the diagnosis of Mycobacterium tuberculosis infection in children correlates with age and immune status Pediatr Infect Dis J 2009 28 8 669 673 10.1097/INF.0b013e3181a16394 19633512

[B9] Connell T Tebruegge M Ritz N Curtis N Interferon-gamma release assays for the diagnosis of tuberculosis Pediatr Infect Dis J 2009 28 8 758 759 10.1097/INF.0b013e3181b00dbf 19633530

[B10] Powell DA Interferon gamma release assays in the evaluation of children with possible Mycobacterium tuberculosis infection: a view to caution Pediatr Infect Dis J 2009 28 8 676 677 10.1097/INF.0b013e3181ad5490 19593249

[B11] Connell TG Tebruegge M Ritz N Bryant PA Leslie D Curtis N Indeterminate interferon-gamma release assay results in children Pediatr Infect Dis J 2010 29 3 285 286 10.1097/INF.0b013e3181c4822f 20190616

[B12] Davies MA Connell T Johannisen C Wood K Pienaar S Wilkinson KA Wilkinson RJ Zar HJ Eley B Beatty D Detection of tuberculosis in HIV-infected children using an enzyme-linked immunospot assay Aids 2009 23 8 961 969 10.1097/QAD.0b013e32832956ad 19287300 PMC4849554

[B13] Liebeschuetz S Bamber S Ewer K Deeks J Pathan AA Lalvani A Diagnosis of tuberculosis in South African children with a T-cell-based assay: a prospective cohort study Lancet 2004 364 9452 2196 2203 10.1016/S0140-6736(04)17592-2 15610806

[B14] Pathan AA Wilkinson KA Klenerman P McShane H Davidson RN Pasvol G Hill AV Lalvani A Direct ex vivo analysis of antigen-specific IFN-gamma-secreting CD4 T cells in Mycobacterium tuberculosis-infected individuals: associations with clinical disease state and effect of treatment J Immunol 2001 167 9 5217 5225 11673535 10.4049/jimmunol.167.9.5217

[B15] Winslow GM Roberts AD Blackman MA Woodland DL Persistence and turnover of antigen-specific CD4 T cells during chronic tuberculosis infection in the mouse J Immunol 2003 170 4 2046 2052 12574375 10.4049/jimmunol.170.4.2046

[B16] Lalvani A Counting antigen-specific T cells: a new approach for monitoring response to tuberculosis treatment? Clin Infect Dis 2004 38 5 757 759 10.1086/381763 14986263

[B17] Chee CB KhinMar KW Gan SH Barkham TM Pushparani M Wang YT Latent tuberculosis infection treatment and T-cell responses to Mycobacterium tuberculosis-specific antigens Am J Respir Crit Care Med 2007 175 3 282 287 10.1164/rccm.200608-1109OC 17082492

[B18] Higuchi K Harada N Mori T Interferon-gamma responses after isoniazid chemotherapy for latent tuberculosis Respirology 2008 13 3 468 472 10.1111/j.1440-1843.2008.01244.x 18399875

[B19] Wilkinson KA Kon OM Newton SM Meintjes G Davidson RN Pasvol G Wilkinson RJ Effect of treatment of latent tuberculosis infection on the T cell response to Mycobacterium tuberculosis antigens J Infect Dis 2006 193 3 354 359 10.1086/499311 16388482

[B20] Aiken AM Hill PC Fox A McAdam KP Jackson-Sillah D Lugos MD Donkor SA Adegbola RA Brookes RH Reversion of the ELISPOT test after treatment in Gambian tuberculosis cases BMC Infect Dis 2006 6 66 10.1186/1471-2334-6-66 16573826 PMC1562425

[B21] Bosshard V Roux-Lombard P Perneger T Metzger M Vivien R Rochat T Janssens JP Do results of the T-SPOT.TB interferon-gamma release assay change after treatment of tuberculosis? Respiratory medicine 2009 103 1 30 34 10.1016/j.rmed.2008.09.012 18977647

[B22] Carrara S Vincenti D Petrosillo N Amicosante M Girardi E Goletti D Use of a T cell-based assay for monitoring efficacy of antituberculosis therapy Clin Infect Dis 2004 38 5 754 756 10.1086/381754 14986262

[B23] Dominguez J De Souza-Galvao M Ruiz-Manzano J Latorre I Prat C Lacoma A Mila C Jimenez MA Blanco S Maldonado J T-cell responses to the Mycobacterium tuberculosis-specific antigens in active tuberculosis patients at the beginning, during, and after antituberculosis treatment Diagn Microbiol Infect Dis 2009 63 1 43 51 10.1016/j.diagmicrobio.2008.09.010 19026511

[B24] Ferrand RA Bothamley GH Whelan A Dockrell HM Interferon-gamma responses to ESAT-6 in tuberculosis patients early into and after anti-tuberculosis treatment Int J Tuberc Lung Dis 2005 9 9 1034 1039 16158897

[B25] Katiyar SK Sampath A Bihari S Mamtani M Kulkarni H Use of the QuantiFERON-TB Gold In-Tube test to monitor treatment efficacy in active pulmonary tuberculosis Int J Tuberc Lung Dis 2008 12 10 1146 1152 18812044

[B26] Kobashi Y Mouri K Yagi S Obase Y Miyashita N Oka M Transitional changes in T-cell responses to Mycobacterium tuberculosis-specific antigens during treatment J Infect 2009 58 3 197 204 10.1016/j.jinf.2008.08.009 18848730

[B27] Kobashi Y Mouri K Yagi S Obase Y Miyashita N Okimoto N Matsushima T Kageoka T Oka M Clinical evaluation for diagnosing active TB disease and transitional change of two commercial blood tests Scand J Infect Dis 2008 40 8 629 634 10.1080/00365540801932454 18642159

[B28] Pai M Joshi R Bandyopadhyay M Narang P Dogra S Taksande B Kalantri S Sensitivity of a whole-blood interferon-gamma assay among patients with pulmonary tuberculosis and variations in T-cell responses during anti-tuberculosis treatment Infection 2007 35 2 98 103 10.1007/s15010-007-6114-z 17401714 PMC2951985

[B29] Ulrichs T Anding R Kaufmann SH Munk ME Numbers of IFN-gamma-producing cells against ESAT-6 increase in tuberculosis patients during chemotherapy Int J Tuberc Lung Dis 2000 4 12 1181 1183 11144463

[B30] Wu-Hsieh BA Chen CK Chang JH Lai SY Wu CH Cheng WC Andersen P Doherty TM Long-lived immune response to early secretory antigenic target 6 in individuals who had recovered from tuberculosis Clin Infect Dis 2001 33 8 1336 1340 10.1086/323044 11565073

[B31] Ewer K Millington KA Deeks JJ Alvarez L Bryant G Lalvani A Dynamic antigen-specific T-cell responses after point-source exposure to Mycobacterium tuberculosis Am J Respir Crit Care Med 2006 174 7 831 839 10.1164/rccm.200511-1783OC 16799072

[B32] Herrmann JL Belloy M Porcher R Simonney N Aboutaam R Lebourgeois M Gaudelus J De Losangeles L Chadelat K Scheinmann P Temporal dynamics of interferon gamma responses in children evaluated for tuberculosis PLoS ONE 2009 4 1 e4130 10.1371/journal.pone.0004130 19125189 PMC2607538

[B33] Nicol MP Pienaar D Wood K Eley B Wilkinson RJ Henderson H Smith L Samodien S Beatty D Enzyme-linked immunospot assay responses to early secretory antigenic target 6, culture filtrate protein 10, and purified protein derivative among children with tuberculosis: implications for diagnosis and monitoring of therapy Clin Infect Dis 2005 40 9 1301 1308 10.1086/429245 15825033

[B34] Hesseling AC Cotton MF Jennings T Whitelaw A Johnson LF Eley B Roux P Godfrey-Faussett P Schaaf HS High incidence of tuberculosis among HIV-infected infants: evidence from a South African population-based study highlights the need for improved tuberculosis control strategies Clin Infect Dis 2009 48 1 108 114 10.1086/595012 19049436

[B35] Millington KA Innes JA Hackforth S Hinks TS Deeks JJ Dosanjh DP Guyot-Revol V Gunatheesan R Klenerman P Lalvani A Dynamic relationship between IFN-gamma and IL-2 profile of Mycobacterium tuberculosis-specific T cells and antigen load J Immunol 2007 178 8 5217 5226 17404305 10.4049/jimmunol.178.8.5217 PMC2743164

[B36] Mantegani P Piana F Codecasa L Galli L Scarpellini P Lazzarin A Cirillo D Fortis C Comparison of an in-house and a commercial RD1-based ELISPOT-IFN-gamma assay for the diagnosis of Mycobacterium tuberculosis infection Clin Med Res 2006 4 4 266 272 10.3121/cmr.4.4.266 17210976 PMC1764813

